# Static and Dynamic Characteristics of a Long-Span Cable-Stayed Bridge with CFRP Cables

**DOI:** 10.3390/ma7064854

**Published:** 2014-06-23

**Authors:** Xu Xie, Xiaozhang Li, Yonggang Shen

**Affiliations:** Department of Civil Engineering, Zhejiang University, Hangzhou 310058, Zhejiang, China; E-Mails: xiexu@zju.edu.cn (X.X.); 11012018@zju.edu.cn (X.L.)

**Keywords:** CFRP stay cables, long span, cable-stayed bridge, static characteristics, dynamic characteristics

## Abstract

In this study, the scope of CFRP cables in cable-stayed bridges is studied by establishing a numerical model of a 1400-m span of the same. The mechanical properties and characteristics of CFRP stay cables and of a cable-stayed bridge with CFRP cables are here subjected to comprehensive analysis. The anomalies in the damping properties of free vibration, nonlinear parametric vibration and wind fluctuating vibration between steel cables and CFRP cables are determined. The structural stiffness, wind resistance and traffic vibration of the cable-stayed bridge with CFRP cables are also analyzed. It was found that the static performances of a cable-stayed bridge with CFRP cables and steel cables are basically the same. The natural frequencies of CFRP cables do not coincide with the major natural frequencies of the cable-stayed bridge, so the likelihood of CFRP cable-bridge coupling vibration is minuscule. For CFRP cables, the response amplitudes of both parametric vibration and wind fluctuating vibration are smaller than those of steel cables. It can be concluded from the research that the use of CFRP cables does not change the dynamic characteristics of the vehicle-bridge coupling vibration. Therefore, they can be used in long-span cable-stayed bridges with an excellent mechanical performance.

## 1. Introduction

Cable-supported bridges have undergone rapid development over the last century since high-strength steel cables were first made available. Cable-stayed bridges can reach spans of over 1000 m, which makes them practical alternatives to suspension bridges. The cables, which transmit most of the force, are key to the structural stability and safety of cable-stayed bridges. However, cables are very vulnerable to corrosion and fatigue damage, which affect their service lifespan, necessitating their replacement. In China alone, the cables of more than twenty bridges have been replaced over the past twenty years for safety reasons [1]. Cable replacement not only requires a huge financial investment, but also disrupts transportation and causes problems in the maintenance and management of the bridge.

Carbon fiber-reinforced plastic (CFRP) has been widely used in civil engineering, because of its light weight, great strength, considerable flexibility and resistance to corrosion and fatigue. In spite of the disadvantages, like the lack of ductility, high cost, contractor unfamiliarity, susceptibility to impact damage and difficulty in forming connections, CFRP cables are superior to steel cables in some critical mechanical aspects, such as creep and relaxation. The possibility of using CFRP in long-span bridges has received considerable attention for this reason. Meier *et al*. [2] evaluated the applicability of CFRP materials in long-span cable-supported bridges. Later, other scholars made more comprehensive studies on the possibility of applying CFRP in long-span bridges [3,4,5,6,7,8,9,10,11,12,13,14]. The mechanical properties of long-span cable-supported bridges built with CFRP cables have been gradually explained. Better ways of manufacturing CFRP cables and anchoring systems have been developed, and these have already seen use in some real-world bridges [15,16,17,18,19]. The increase in knowledge of CFRP materials has decreased the manufacturing costs, and the use of CFRP cables in long-span bridges will upsurge in the near future.

So far, most studies of CFRP cables have been limited in scientific premise. Some problems are still at the probing stage. The impact of CFRP cables on the mechanical properties of long-span cable-stayed bridges has not yet been subjected to systematic research. In this research, a series of studies have been carried out on CFRP cables in bridges of different sizes. Herein, a CFRP cable-stayed bridge is compared to a steel cable-stayed bridge with respect to material, element and structural characteristics. These results provide evidence supporting the use of CFRP cables in long-span cable-stayed bridges.

## 2. Mechanical Properties of CFRP Cables

### 2.1. Material Properties of CFRP Cables

At present, there is no uniform production standard for CFRP cables, so the material parameters of CFRP cables produced by different manufacturers differ considerably. Table 1 shows the material properties of four types of CFRP cables, marked as A, B, C and D. In the table, γ, *E*, σ_u_, ε_u_, *R*_e_ and ρ represent the unit weight, Young’s modulus, tensile strength, ultimate strain, relaxation ratio for 1000 h and linear expansion coefficient, respectively. It was here observed that the material characteristics of CFRP cables produced by different manufacturers are typically similar. The Young’s modulus is about 140 GPa, which is about 70% of steel cables. The tensile strength is 2.02–2.55 GPa, which is 1.3–1.6 times that of high-strength steel cables. The linear expansion coefficient is 0.6 × 10^−6^/°C, and the temperature deformation is only 1/20 that of steel cables. The unit weight is 1/5 that of steel cables, and the relaxation rate is lower than that of steel cables. In addition, the ultimate strain of CFRP cable is so small that the stress-strain curve remains mostly linear before fissure, which indicates that CFRP cables are brittle.

**Table 1:** Material properties of CFRP and steel cables.

Cable types	γ (kN/mm^3^)	σ_u_ (MPa)	*E* (GPa)	σ_u _/*E*	*R*_e_ (%)	ε_u_ (%)	*a*_l _(×10^−6^/°C)
A	16	2,140	137	0.016	0.3	1.6	0.6
B	16	2,550	147	0.017	0.3	1.6	0.68
C	16	2,022	137	0.015	0.3	2	0.6
D	16	2,421	159	0.015	0.3	1.7	0.6
steel	77	1,570	196	0.008	<2.5	>4	12

### 2.2. Damping Properties of CFRP Cables

Cable damping is mainly associated with energy dissipation caused by material strain and air friction. There is so little air damping that it can usually be ignored during calculation. This paper focuses on the material damping characteristics of CFRP cables. According to the strain energy proportional damping theory of a single-degree-of-freedom vibration system, the damping ratio ξ can be calculated using the following expression:

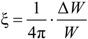
(1)
Here, Δ*W* is the energy absorbed due to the strain deformation during the vibration cycle, and *W* is the modal potential energy. Assuming Δ*W* is proportional to the strain energy Δ*W*∝*V*, replacing the proportionality sign with a constant [20,21,22]:

Δ*W* = 2πη*V* (2)

Here, η is the energy loss factor, and *V* is the strain energy. Substitution of the equation above into Equation (1) produces the following:

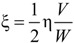
(3)


Because there is a large initial tension *T*_0_ in the cable, therefore the modal potential energy *W* of Formula (3) includes two parts, initial tension potential energy *W*_0_ and strain energy *V*, hence:

*W* = *W*_0_ + *V* (4)

**Figure 1:**
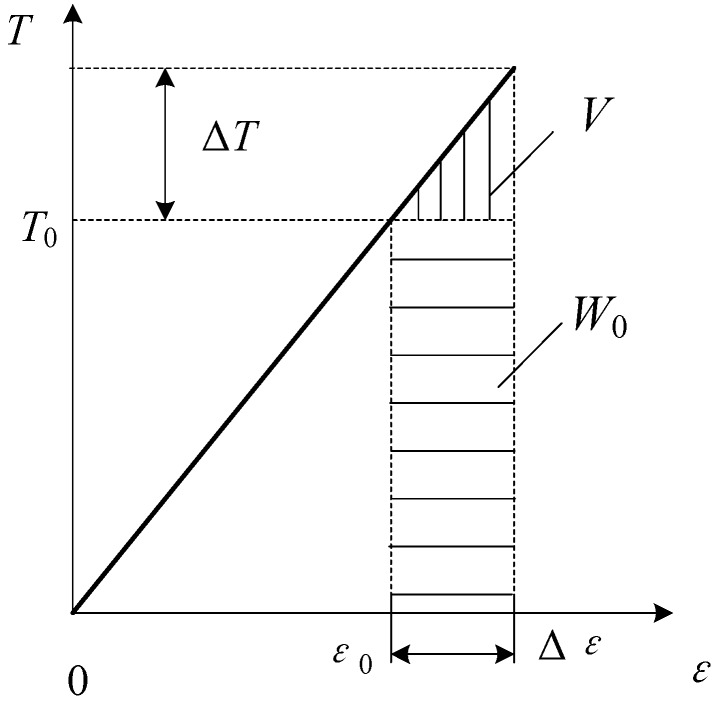
Potential energy and strain energy in initial tension.

Because the flexible cable only bears axial tension, as indicated by the relationship of strain and tension shown in Figure 1, the initial potential energy due to tension, *W*_0_ and strain energy *V* can be calculated as follows:

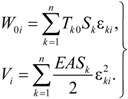
(5)


Here, ε*_ki_* and *S_k_* are the strain and length of element *k* generated by the *i-*th vibration mode. *T_k_*_0_ is the initial tension in element *k*, and *n* is the total number of cable elements. Substitution of Equations (4) and (5) into (1) results in the following expression:

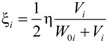
(6)


**Figure 2:**
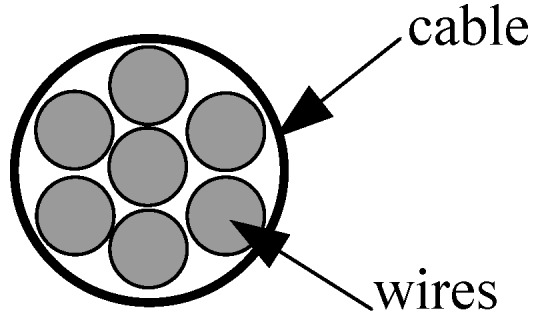
Cross-section of cable CFCC*ϕ*12.5.

In order to compare the damping characteristics of steel and CFRP cables, a cable vibration test was carried out in Tokyo Metropolitan University [22]. The test steel and CFRP cables were made at Rope Corporation Tokyo, Japan, wherein the steel cable, PCS*ϕ*12.7 (*ϕ* refers to diameter of the cable), was composed of seven 5.16-mm diameter steel wires, PCS*ϕ*5.16, and the CFRP cable, CFCC*ϕ*12.5, was composed of seven 5.0 mm-diameter CFRP wires, CFCC*ϕ*5.0 (Figure 2). Table 2 lists the material parameters of cables and wires. *E*, *w*, *D*, *A* and σ_u_ represent Young’s modulus, the unit weight, the diameter, the cross-sectional area and the tensile strength of cables and wires, respectively.

**Table 2:** Material properties of cables and wires.

Cable and Wire Type	*E* (GPa)	*w* (g/m)	*D* (mm)	*A* (mm^2^)	σ_u_ (MPa)
CFCC*ϕ*12.5	159	145	12.50	76.0	2,421
PCS*ϕ*12.7	197	774	12.70	98.7	1,854
CFCC*ϕ*5.0	152	29.9	5.00	15.2	2,980
PCS*ϕ*5.16	204	163.8	5.16	20.9	1,999

**Figure 3:**
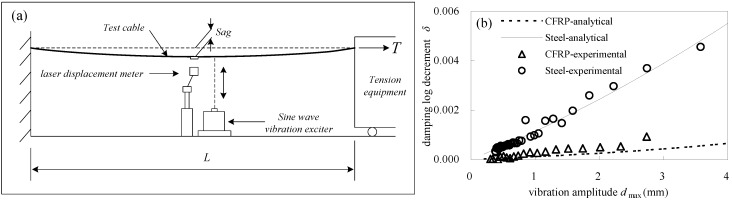
(**a**) Cabledamping test; (**b**) cable damping log decrement *vs*. vibration amplitude.

Figure 3a,b provides details of the cable damping test equipment. The test cable was 20 m-long with an initial tension of 30 kN. The resulting initial stresses in CFRP cables and steel cables were 395 and 304 MPa, respectively, which were equal to half of the service stresses. Here, the design safety factor of the test cable was 2.5. Continuous excitation force was applied at the midpoint of the cable to render the vibrations stable. Once the excitation force ceased, the cable entered free decay vibration, which was measured by a non-contact laser displacement meter at a sampling rate of 500 Hz. Figure 3b shows the relationship between the cable damping and vibration amplitude. It can be found that, for steel and CFRP cables, damping increases with vibration amplitude, but the trend was more obvious for steel cables than CFRP cables. This indicted that the influence of vibration amplitude on cable damping is not as pronounced for CFRP cable as it is for the steel cable. In addition, the energy loss factor of cables shown in Equation (3) can be calculated from Figure 3b. Here, the damping constant was 0.05 for both steel and CFRP cables, consistent with the test results reported by Kady *et al.* [23]. 

**Figure 4:**
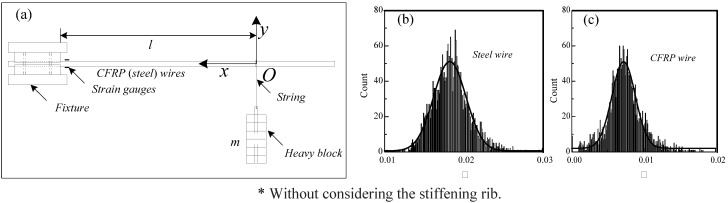
(**a**) Bending deformation experiments of steel and CFRP wires; (**b**) distribution of energy loss coefficient for steel wires; (**c**) distribution of energy loss coefficient for CFRP wires.

In order to determine the energy loss factors of wires comprising the cables, the bending deformation experiment of steel and CFRP wires under free vibration was conducted (Figure 4a. A heavy block was hung on by a string on the fixed test wire at a distance of *l* from the fixed end. An initial imposed displacement was used to place the wire in decay free vibration. The strain readings were recorded by the dynamic strain gauges fixed on the wire, at a sampling of 500 Hz. During the test, the distance *l* and the mass of the heavy block were varied multiple times to obtain additional experimental data.

Figure 4b shows the distribution results of energy loss factors for steel and CFRP wires. As shown, the average energy loss factors for steel and CFRP wires are about 0.018 and 0.008, respectively, which is much smaller than the damping constant of steel and CFRP cables, 0.05. This indicates that the damping of the material strain ratio is only a part of the total cable damping, and other damping is relative to the friction among the wires of the cable.

## 3. Structural Description of the Cable-Stayed Bridge

**Figure 5:**
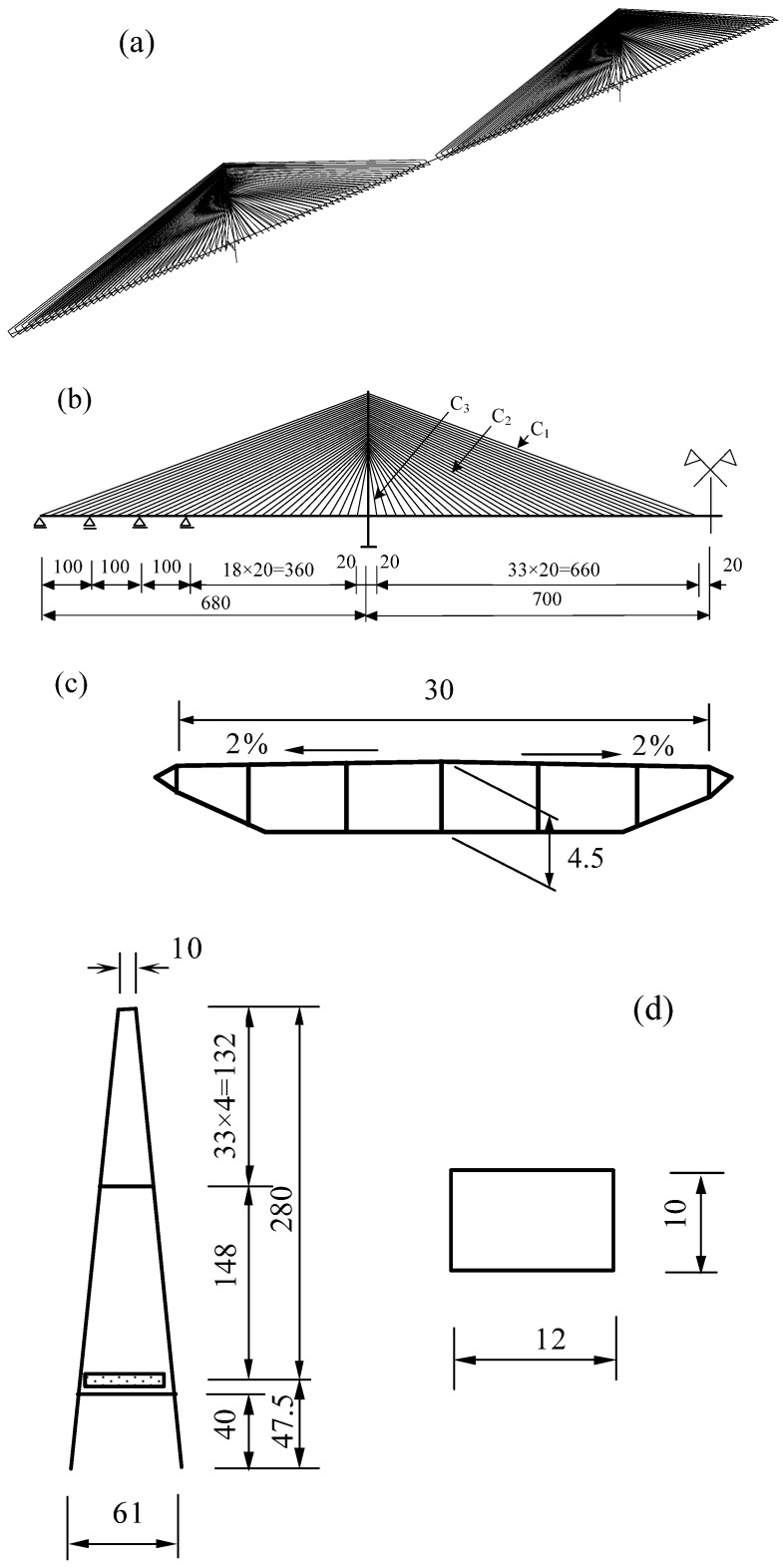
(**a**) 3D model of the bridge; (**b**) layout of half of the bridge; (**c**) typical cross-section of the box girder; (**d**) layout of the pylon and cross-section of the tower.

In this paper, the mechanical characteristics of a cable-stayed bridge with CFRP cables are discussed, and the feasibility of using CFRP cables on long-span cable-stayed bridges is evaluated. The finite element method (FEM) is used to establish a numerical model of the 1400-m span of a cable-stayed bridge. Although the bridge model is not an actual structure, the geometric parameters were chosen by a conceptual design procedure according to some basic parameters of the existing long-span cable-stayed bridges [22]. Figure 5 shows the layout of the bridge model (in meters). This bridge’s mid-span is 1400 m, which is about two times its side-span. Thirty-four cables are installed at a horizontal spacing of 20 m on each side of towers to hold the main beam, which is a uniform multi-cell flat steel box girder. The width of the box girder is 30 m, and the height is 4.5 m, which indicates a relatively larger height-to-span ratio as compared to many other cable-stayed bridges. The equivalent thicknesses of the orthotropic top and bottom plates of the girder are 20 mm (derived as 12 mm of steel plate and an 8-mm equivalent thickness of the stiffener). The equivalent thickness of the web is 15 mm. The height of the pylon is 280 m, which is 0.2-times the mid-span, and the tower cross-section is a single cell box composed of walls with an equivalent thickness of 40 mm. Table 3 lists the cross-sectional parameters of the girder and the tower. Along the side-span, there are three middle auxiliary piers at a horizontal spacing of 100 m to support the girder. Constraint springs were set between the girder and the pylon along the bridge longitudinal axis. Cables of C1, C2 and C3, shown in Figure 5, represent long, medium and short cables of Type C, mentioned in Table 1. The cables here have a relatively low tensile strength of 2022 MPa and an elastic modulus of 137 kN/mm^2^. In the numerical model, the tower and box girder are 1111 spatial beam elements with 6 DOF, and the cables are 272 isoparametric elements with 3 DOF. The bridge is fixed at the bottom of the tower and supported by piers. The bridge can move along the longitudinal axis. The geometric nonlinear method and material nonlinear method are used to solve the problem.

The unit weight of the structure is calculated as follows:

*W*_D_ = *k*_D_γ*A* + *W*_0_ (7)

Here, *k*_D_ is the load factor, chosen by conceptual design considering the self-weight of the stiffener plate of the box girder. [22]. For girders *k*_D_ = 1.4, and for pylons, *k*_D_ = 1.2. γ is the unit weight of steel. *A* is the cross-sectional area of box girder. *W*_0_ is the secondary load, which mainly consists of pavement load, *etc.*, taken as, *W*_0_ = 70 kN/m. The secondary load of the pylon is relatively so small, that it can be ignored.

**Table 3:** Cross-sectional properties.

Cross-Section	*A* (m^2^)	*I_x_* (m^4^)	*I_y_* (m^4^)	Torsion Constant (m^4^)
Girder	1.607	5.503	120.43	10.352 *
Tower	1.760	30.667	40.320	39.273 *

* Without considering the stiffening rib.

The cross-sectional area of the cable is calculated using the maximum cable tension *T*_0_ and the allowable stress σ. The formula is as follows:

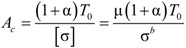
(8)
Here, α is the factor of proportionality. It indicates how much cable tension is produced by the live load in response to the maximum tension produced by the dead load in the long-span cable-stayed bridge. Because the factor of proportionality for long-span cable-stayed bridges is usually not more than 0.2, it is here assumed that α = 0.2. σ is the allowable cable stress, and σ^b^ is the standard value of tensile strength; μ is the safety factor for the cable, which is generally 2.5 for steel cables. It is here assumed that μ = 3.0 for CFRP cables, because CFRP is a strong, but brittle, material.

Table 4 shows the cable self-weight for steel cable-stayed bridges and CFRP cable-stayed bridges of the same span. Results show that the self-weight of the CFRP cable is only about 1/7 that of steel cable, which is less than the unit weight ratio of CFRP to steel, which is about 1/5. This means that fewer CFRP than steel cables would be needed for a cable-stayed bridge. This is because CFRP cables sag less, giving them greater working efficiency. This may substantiate the cost disadvantage of CFRP cables.

**Table 4:** Weight of steel and CFRP cables on a whole bridge.

Material	Weight (kN)	Weight Ratio of Steel to CFRP
Steel	115,133	6.8
CFRP	17,056.7	

## 4. Dynamic Characteristics of CFRP Stay Cables

### 4.1. Natural Vibration Characteristics

**Figure 6:**
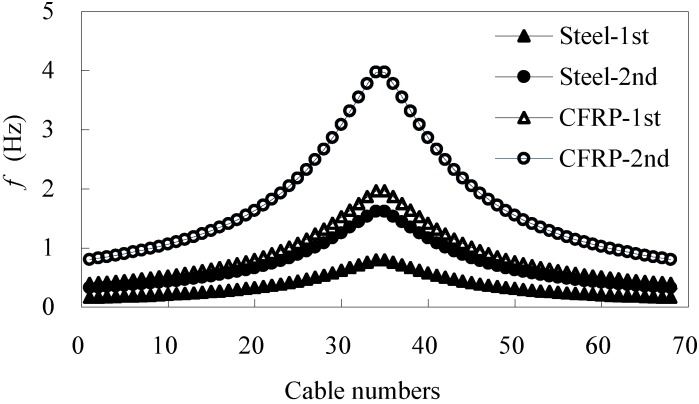
Natural frequencies of steel and CFRP cables.

Figure 6 gives the first two order natural frequencies of cables on one side of the half bridge shown in Figure 5. Because there is little difference between the in-plane and out-of-plane vibration, these results only demonstrate the in-plane vibration results. In the figure, the vertical axis *f* is the natural frequency, and the cable numbers on the horizontal axis represent the position of the cable anchor on the girder expressed in terms of the number of markers from the start of the side-span to the midpoint of the bridge. The results show that, due to the large self-weight, the natural frequencies of steel cables are about 0.5-times those of CFRP cables.

The material properties and natural frequencies of stay cables of three different lengths (marked C1, C2 and C3 in Figure 5) are compared in Table 5 and Table 6. *S* represents the steel cable, *C* the CFRP cable, *EA* the axial stiffness and *w* the unit weight. The Irvine parameter λ, a dimensionless parameter reflecting the dynamic characteristics, is defined as follows [24]:

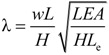
(9)
Here, *H* represents the horizontal component of cable tension and *L*_e_ represents cable length. Expression (9) indicates that the Irvine parameter is related to the geometric and material properties of the cable, the smaller the sag and the tensile stiffness of the cable, the smaller the Irvine parameter. Table 5 shows that, for the same span cables, the Irvine parameters of steel cables are much larger than those of CFRP cables. For cables made of the same material, the Irvine parameters for long cables are larger than that for short cables. Table 6 shows that, for the same cable span, the natural frequencies of CFRP cables are about two-times those of steel cables.

**Table 5:** Material properties of stay cables.

Cables	*L* (m)	*Y* (m)	*EA* (kN)	*W* (kN/m)	λ
S-C1	660	275.5	3,045,840	1.3114	0.01750
S-C2	460	235.5	2,516,640	1.0835	0.01306
S-C3	260	195.5	1,795,360	0.7730	0.00912
C-C1	660	275.5	1,557,416	0.1840	0.002863
C-C2	460	235.5	1,377,507	0.1630	0.002144
C-C3	260	195.5	1,033,802	0.1220	0.001501

**Table 6:** Natural frequencies of stay cables (units: Hz).

Cables	Out-of-Plane		In-Plane	
	1st	2nd	1st	2nd
S-C1	0.1652	0.3343	0.1998	0.3341
S-C2	0.2289	0.4635	0.2532	0.4633
S-C3	0.3645	0.7379	0.3771	0.7378
C-C1	0.4083	0.8267	0.4098	0.8266
C-C2	0.5662	1.1443	0.5652	1.1443
C-C3	0.8986	1.8194	0.8991	1.8194

In order to analyze the relationship between the cables and the dynamics of the bridge itself with respect to natural frequencies, the major frequencies of the bridge were calculated taking the influence of local vibrations of the cable into account (Table 7). The results show that the natural frequencies of steel cable-stayed bridges are basically the same as those of CFRP cable-stayed bridges. 

**Table 7:** Major natural frequencies of cable-stayed bridge (units: Hz).

Cable Type	First Lateral Bending	Longitudinal Drifting	First Vertical Bending
Steel cables	0.0319	0.0330	0.1440
CFRP cables	0.0324	0.0340	0.1370

**Figure 7:**
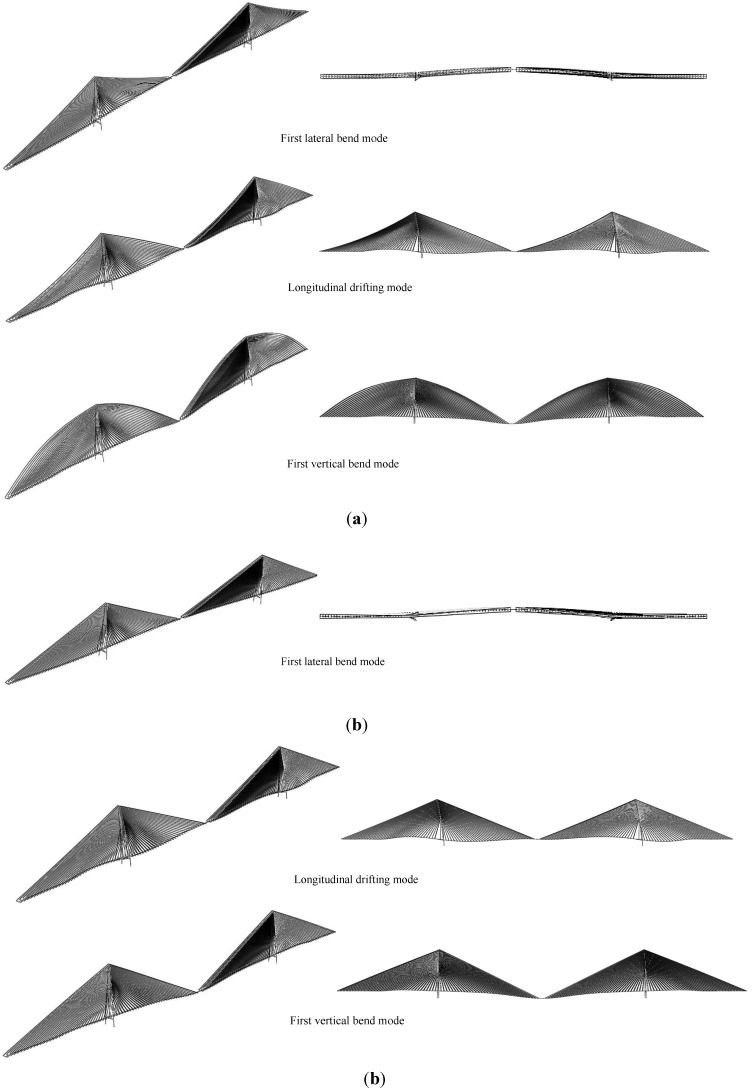
(**a**) Vibration modes of cable-stayed bridges with steel cables; (**b**) vibration modes of cable-stayed bridges with CFRP cables.

It can be found from Table 6 and Table 7 that the natural frequencies of steel cables overlap those of the first vertical bending frequencies of steel cable-stayed bridge. However, the natural frequencies of CFRP cables differ from the low natural frequencies of the CFRP cable-stayed bridge. That is why the steel cable-bridge coupling vibrations occur so easily. Therefore, using CFRP cables can significantly reduce the possibility of coupling vibrations in the cable bridge. Some major vibration modes of steel cable-stayed bridges and CFRP cable-stayed bridges are shown in Figure 7. It was found that the local vibrations of the cables in steel cable-stayed bridges were dominant for the first vertical bending vibration.

### 4.2. Damping Characteristics

**Figure 8:**
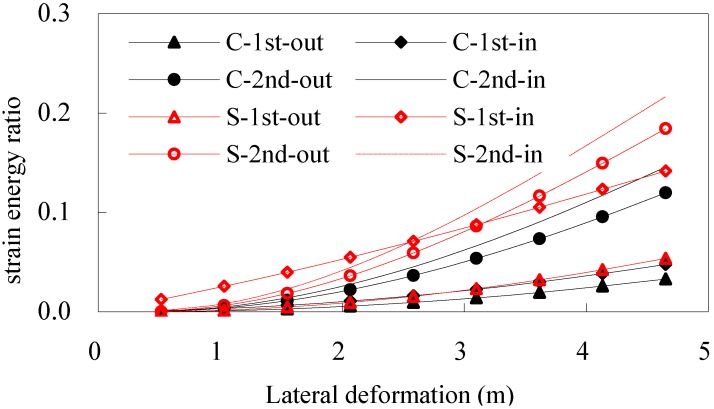
Strain energy ratios of steel and CFRP cables.

Because the strain energy ratios of different lengths of cables are similar, only cable C2 is analyzed in this paper. Figure 8 shows the relationship between the first two vibration modal strain energy ratios calculated using Formulas (5) and (6) and the cable lateral in-plane and out-of-plane amplitudes. In the figure, S represents the steel cable, C represents the CFRP cable, -1st and -2nd represent the first and the second vibration modes and -out and -in represent the in-plane and out-of-plane vibration modes. The results show that the modal strain energy ratios of two materials are on the same order of magnitude. CFRP cables showed lower strain energy ratios than steel cables. If the vibration amplitude is around 1 m, Formula (6) can be used to calculate the structural damping ratios of both steel and CFRP cables, which were here around 1%.

**Figure 9:**
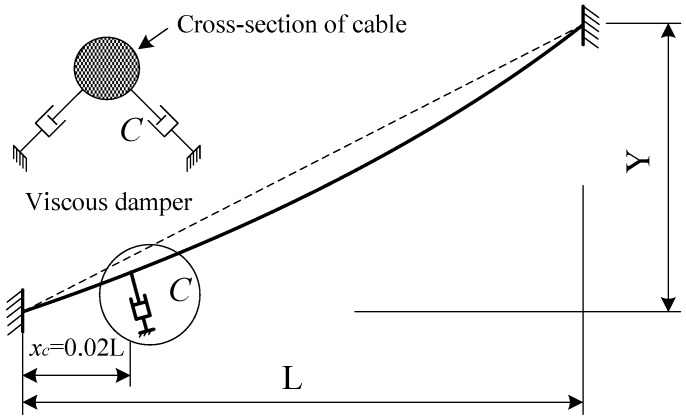
Stay cable with viscous damper.

The external viscous damper is usually arranged at the lower end of the cable in order to prevent large vibration. Figure 9 shows the layout of the cable with the external viscous damper. *x_c_* is the horizontal distance between the lower end of the cable and the damper. In this paper, *x_c _*= 0.02 L. The non-proportional damping calculation theory (complex eigenvalue method) was used to calculate the damping value.

**Figure 10:**
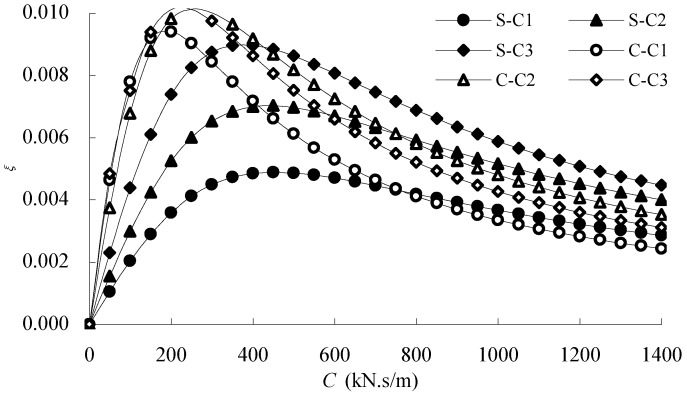
Effects of external dampers on modal damping.

The calculation results show that the damping coefficients *C* have little impact on the natural frequencies of the steel and CFRP cables. Figure 10 shows the relationship between the first in-plane modal damping ratios and the damping coefficients. The damping coefficients significantly affected the modal damping ratios. As the damping coefficients increase, the modal damping ratios also increase significantly, peak and then drop quickly. There is an optimal range within which the damping coefficients produce desirable effects. The optimal damping coefficient can be found using the following formula [25].

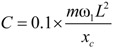
(10)
Here, *m* represents the unit mass and ω_1_ represents the in-plane damped natural circular frequency of the first mode of the stay cable. Comparisons showed that the optimal viscous damping coefficients of CFRP cables were smaller than that of steel cables. This shows that only smaller dampers can produce the similar effects on CFRP cables that have been seen on steel cables.

### 4.3. Parametric Vibration Characteristics of CFRP Stay Cables

#### 4.3.1. Calculation Model and Method

Parametric vibrations are serious hindrances for stay cables, and analyzing this aspect has drawn considerable attention [26,27,28,29,30,31,32,33,34]. Three steel and three CFRP cables were used to compare the differences in parametric vibration characteristics (Table 5). The impact of the external viscous damper on parametric vibration was also studied. The nonlinear finite element method was used to calculate the parametric vibration of the stay cables [35,36]. The structural damping was ignored in the calculation process.

**Figure 11:**
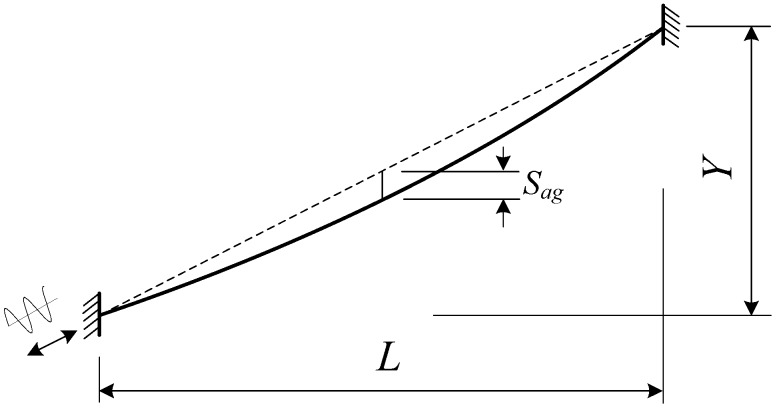
Parametric vibration analysis model of the stay cable.

Figure 11 gives the model of stay cable parametric vibration analysis. A harmonic excitation vibration in the chord direction is given at the lower end of the cable. The vibration amplitude is 1/50,000 of the cable span. The excitation frequency is taken as *f* = *f*_1_ and *f* = 2*f*_1_. Here, *f*_1_ is the in-plane natural frequency of the first mode.

#### 4.3.2. Parametric Vibration of Stay Cables Undamped

**Figure 12:**
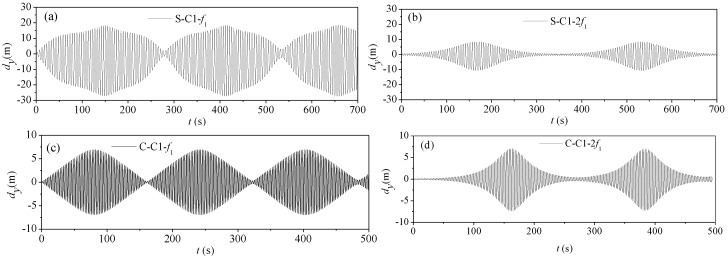
Parametric vibration response of stay cable C1 (undamped): (**a**) steel cable under excitation frequency *f* = *f*_1_; (**b**) steel cable under excitation frequency *f* = 2*f*_1_; (**c**) CFRP cable under excitation frequency *f* = *f*_1_; (**d**) CFRP cable under excitation frequency *f* = 2*f*_1_.

Figure 12 gives the time history curves of vibration responses at the midpoint of the stay cable C1 when the excitation frequencies are *f* = *f*_1_ and *f* = *f*_2_, respectively. As shown, the parametric vibration response of the CFRP cable is weaker than that of the steel cable. It also requires more time to excite a larger amplitude for the CFRP cable. This indicates that, to some extent, the use of CFRP cables involves lesser parametric vibration than that of steel cables.

Table 8 gives the parametric vibration response amplitudes of stay cables C1, C2 and C3. It shows that, regardless of the length of the cable, the response amplitude of CFRP cables is weaker than that of steel cables.

**Table 8:** Response amplitudes of parametric vibration (units: m).

Cable	Steel		CFRP	
	*f*_1_	2 *f*_1_	*f*_1_	2 *f*_1_
C1	28.460	10.670	7.059	7.410
C2	11.590	11.150	4.270	4.946
C3	4.007	3.680	1.961	1.961

#### 4.3.3. Parametric Vibration of Damped Stay Cables

To study the difference in the damping effect on the parametric vibration of CFRP and steel cables, the viscous dampers shown in Figure 9 were arranged at the lower end of cable C1. Assuming that *x_c_* = 10 m, two viscous dampers were arranged symmetrically at either side of the cable, and the angle between the two dampers was 60°. The optimal damping coefficient for CFRP cable single damper obtained using Equation (10) is 448.86kN·s/m, is much smaller than that of the steel cable, 127.35 kN·s/m. 

**Figure 13:**
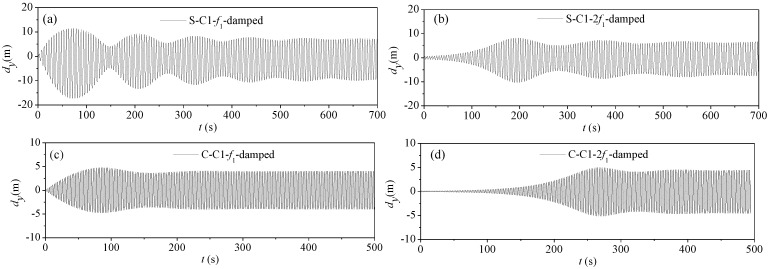
Parametric vibration response of stay cable C1 (damped): (**a**) steel cable under excitation frequency *f* = *f*_1_; (**b**) steel cable under excitation frequency *f* = 2*f*_1_; (**c**) CFRP cable under excitation frequency *f* = *f*_1_; (**d**) CFRP cable under excitation frequency *f* = 2*f*_1_.

Figure 13 gives the time history curves of vibration responses at the midpoint of stay cable C1 damped when the excitation frequencies were *f* = *f*_1_ and *f* = 2*f*_1_, respectively. Figure 12 and Figure 13 show that, although the damper is effective for the cable vibration, it cannot lower the response amplitude after the parametric vibration comes into picture.

### 4.4. Fluctuating Vibration Characteristics of CFRP Stay Cables under Wind Load

#### 4.4.1. Calculation

The analysis of wind loads to which the cables are subjected is very complex. The fluctuating wind load can cause them to vibrate. The turbulence power spectral density function of fluctuating wind is as follows [37]:

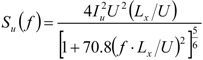
(11)

*L_x_* = 100(*Z*/30)^0.5^ (12)

Here, *S_u_*(*f*) represents the turbulence power spectral density function; *L_x_* represents the scale of turbulence; *I*_u_ represents turbulence intensity, which is related to wind speed, and the ground roughness coefficient *I*_u_ = 0.1 in this paper. *Z* is the distance of the girder from the surface of the water surface. Here, *Z* = 50 m.

The random velocity of fluctuating wind is as follows [38]:

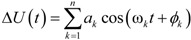
(13)
Here, *ϕ_k_* is the randomly-generated phase angle, and other parameters are calculated as follows:

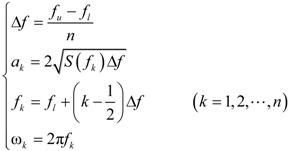
(14)
Here, *f_u_* and *f_l_* are the upper and lower bounds of cable frequencies and *n* is an integer. The power spectral density of fluctuating wind sample with an average speed of 30 m/s was generated using Equation (11).

**Figure 14:**
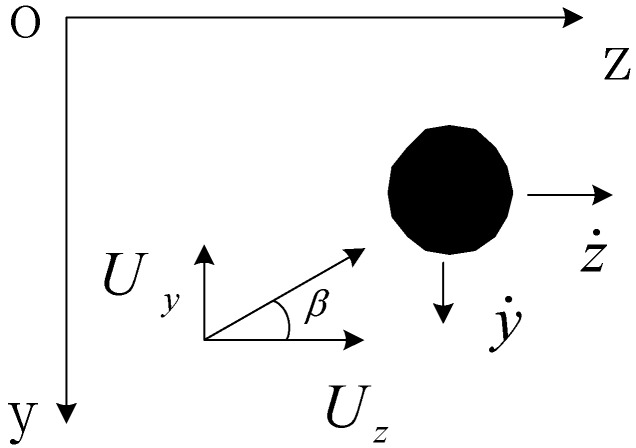
Cross-section of cable under lateral wind load.

For the cable laden by lateral wind load shown in Figure 14, the relative wind attack angle θ can be determined as follows:

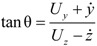
(15)
Here, *U_y_* and *U_z_* are vertical and horizontal components of fluctuating wind speed, which are calculated as follows:

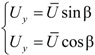
(16)
Here, β is the wind attack angle shown in Figure 14; *ẏ* and *ż* are the vertical and horizontal vibration components, respectively. *U* is the sum of average wind speed and fluctuating wind speed.

The relative wind speed experienced by the cable *U_r_* is as follows:

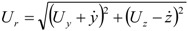
(17)


The drag force *D* and lift force *L* acting on the cable along the relative wind direction are as follows:

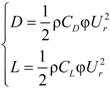
(18)
Here, *C*_D_ and *C*_L_ are the drag and lift coefficients of the cable. *C*_D _= 0.7 and *C*_L _= 0 for the round cross-section of the cable. φ is the outside diameter of the cable. ρ is the air density. The vertical and horizontal wind loads can be compounded by drag and lift force:

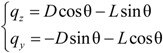
(19)


#### 4.4.2. Wind Fluctuating Vibration of Stay Cables

**Figure 15:**
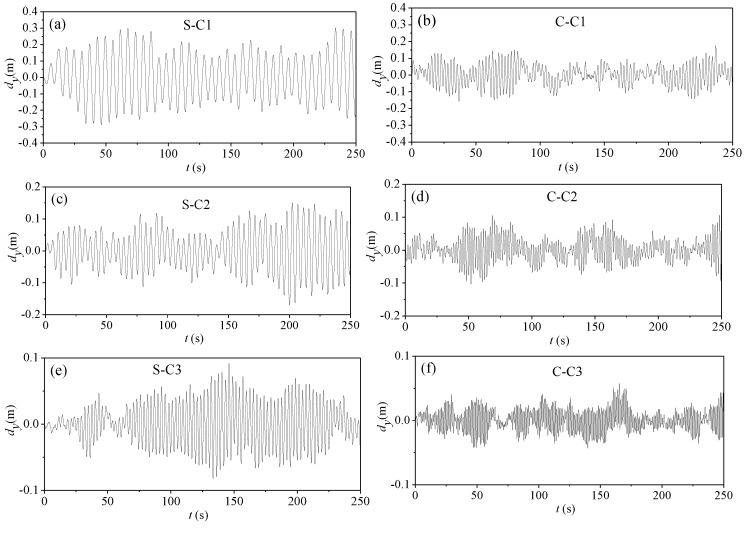
Wind fluctuating vibration responses of stay cables: (**a**) steel cable C1; (**b**) CFRP cable C1; (**c**) steel cable C2; (**d**) CFRP cable C2; (**e**) steel cable C3; (**f**) CFRP cable C3.

Figure 15 gives the fluctuating vibration responses of stay cables C1, C2 and C3 under lateral wind load. As shown, the wind fluctuating vibration responses of CFRP cables are smaller than those of steel cables. The vertical vibration amplitudes of CFRP cables are about half those of steel cables of the same length. This also indicates that bridges built with CFRP cables are less subjected to wind fluctuating vibrations.

## 5. Static and Dynamic Characteristics of Cable-Stayed Bridge with CFRP Cables

### 5.1. Static Characteristics and Stability of Bridge under Live Load

To study the influence of CFRP cables on the mechanical properties of the bridge, the nonlinear finite element method is used to analyze the static characteristics of the girder. The designed distribution load is 43 kN/m, and the designed concentrate load is 1000kN [39]. The nonlinear equation is as follows:

**K**(**u**)**u** = **F** (20)

Here, **u** is the displacement vector; **K**(**u**) is the stiffness matrix considering the initial internal force and geometrical nonlinearity; and **F** is the live load vector. To determine the most unfavorable loading position of the live load, the tangent stiffness at the finished state of the bridge is used to identify the influence line. The reduction of the live load with the span is neglected.

**Figure 16:**
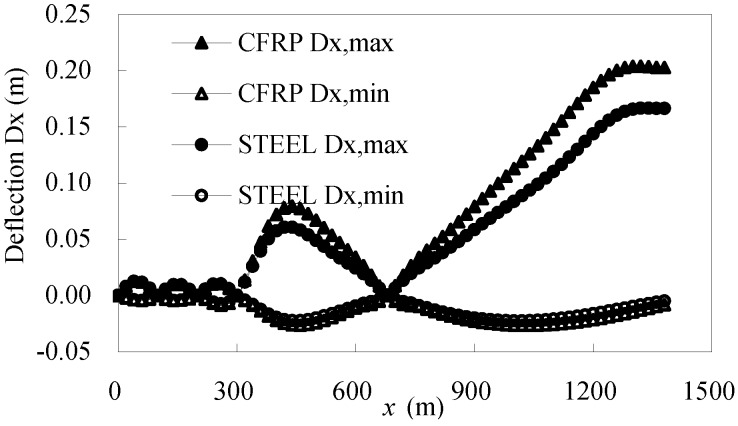
Maximum and minimum girder deflections under live load.

Figure 16 gives the maximum and minimum deflection values of a half box girder. The minimum deflection of the CFRP cable-stayed bridge was nearly same as that of the steel cable-stayed bridge. However, because steel-cable-stayed bridges have greater structural rigidity, the maximum girder deflection is 0.000119 of the span, but for CFRP cable-stayed bridges, it is 0.000146 of the span, which is 1.22-times the former. This indicates that the material properties of the cable have little impact on the minimum girder deflection and considerable impact on the maximum deflection. The critical girder deflection of cable-stayed bridge is 0.001 of the span, meaning that replacing steel cables with CFRP cables might give the bridge a large margin of safety, with respect to structural rigidity.

Under dead and live loads, the maximum compressive stress in the girders of steel-cable-stayed bridges is 189.3 MPa; that of CFRP-cable-stayed bridges it is 9% lower, at 173.9 MPa. This means that CFRP cables may be suitable for long-span cable-stayed bridges.

Structural stability is very important for long-span cable-stayed bridges, because there is a large amount of axial pressure in girders and pylons. The stability safety factor can be obtained using the following complex eigenvalue equation [40]:

det(**K**_ep_ + λ**K**_G_) = 0 (21)

Here, **K**_ep_ is the stiffness matrix, which takes material nonlinearity into consideration; **K**_G_ is the geometric stiffness matrix; and λ is the stability safety factor. Unlike the linear elastic eigenvalue method, the elastic modulus in Equation (21) was obtained based on the law that the buckling strength is equal to the experimental strength, *i.e.*, the effective elastic modulus was adopted for the stiffness matrix, which considers the influence of initial defect and elasto-plastic stress of the structural member.

**Figure 17:**
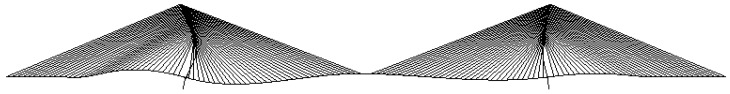
Elasto-plastic buckling mode of cable-stayed bridge.

Elasto-plastic branch instability analysis showed that the minimum stability factor is 3.03 for CFRP cable-stayed bridges and 2.88 for steel cable-stayed bridges. The difference is attributed to the buckling caused primarily by the pylon. The self-weight of the steel cables is greater than that of CFRP cables, so the axial force in the pylon for steel-cable-stayed bridge is greater than that for a CFRP-cable-stayed bridge. Figure 17 gives an example of the elasto-plastic buckling mode for cable-stayed bridge with CFRP cables.

### 5.2. Aerostatic Characteristics of Bridges under Wind Load

Wind load is an important factor in the design of long-span cable-stayed bridges. It has two effects on the structure: aerostatic and aerodynamic. Fluttering causes the greatest damage amongst all other aerodynamic effects. Cable-stayed bridges with streamlined, flat steel box girders are more aerodynamic resistant, giving the bridge a relatively high critical wind speed. Therefore, the present paper focuses mainly on the transverse deformation of the structure under static wind load.

**Figure 18:**
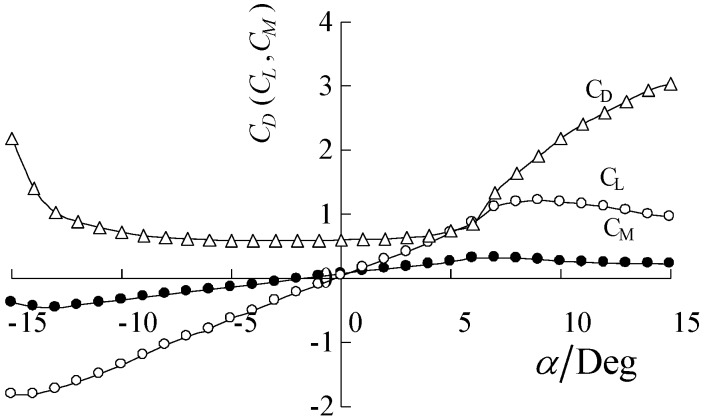
Correlation between aerostatic coefficients and the wind attack angles.

The aerostatic effect on the structure is related to the structure shape and wind attack angle. In this paper, the static wind load is calculated using three aerostatic coefficients based on the wind tunnel test results of scale model. The aerostatic actions on the box girder are as follows:

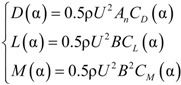
(22)
Here, α is the wind attack angle; ρ is air density; *B* is girder width; *A_n_* is girder height in the windward side; and *U* is wind speed. Here, *U* = 40 m/s. *D*, *L* and *M* are the drag force, the lift force and the torque action on unit length of the box girder in relation to the drag coefficient *C_D_*, the lift coefficient *C_L_* and the torque coefficient *C_M_*, respectively. Figure 18 gives the relation curves between the three aerostatic coefficients and the wind attack angles.

**Figure 19:**
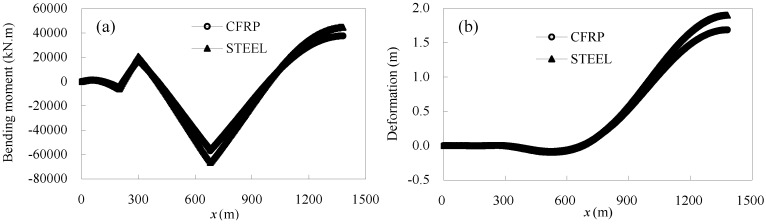
(**a**) Transverse bending moment of girder under wind load; (**b**) transverse deformation of girder under wind load.

Figure 19 gives the transverse bending moment and deformation of the half-bridge girder under the design wind load. Due to the constraints imposed by the middle piers, the bending moment and deformation of the side span girders are very small, but that of the mid-span girder is considerably high. The transverse bending moment and deformation of mid-span girders is slightly larger for steel cable-stayed bridges than that for CFRP cable-stayed bridges. The logic behind this is that the out-of-plane deformation of the girder is related to the windward area of the cable, and steel cables have more windward area than CFRP cables. 

The analysis of the instability of cable-stayed bridges under static wind load showed that when the wind speed reaches 60 m/s for a complete bridge or 50 m/s for a bridge still under construction, bridges built with CFRP cables showed no buckling failure. This indicates that CFRP cable-stayed bridges have better wind resistance than steel cable-stayed bridges.

### 5.3. Dynamic Response of Bridge under Traffic Load

**Figure 20:**
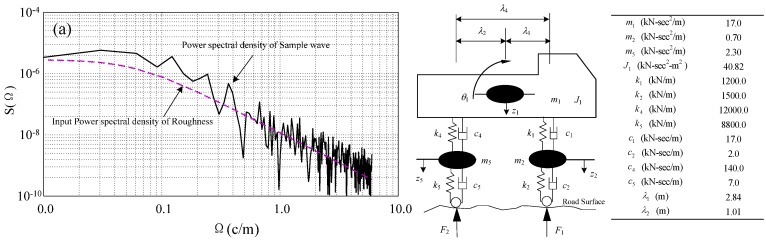
(**a**) Four-degree vehicle model; (**b**) power spectral density of sample wave.

Dynamic response due to traffic loads has a major impact on the safety and durability of a cable-stayed bridge. A vehicle-bridge coupling model was established to study the load impact factor and the cable response of CFRP cable-stayed bridges under traffic load. The girder deflection and cable tension response of the bridge were evaluated for different vehicle speeds and levels of road roughness. Because some of the first natural frequencies of long-span cable-stayed bridges are relatively small, the calculated results of vehicle-bridge coupling using sophisticated vehicle models show little difference from those produced using simplified vehicle models. For this reason, the four-degree vehicle model shown in Figure 20a was used to facilitate calculation. Figure 20b shows the power spectral density of a sample wave that represents the relatively smooth road surface [41].

**Figure 21:**
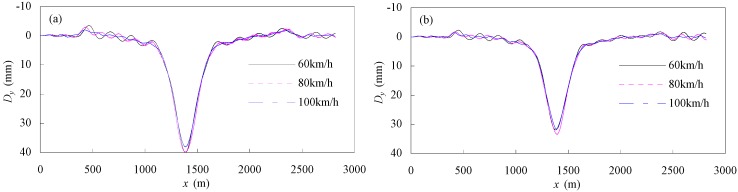
(**a**) Deflection response of CFRP cable-stayed bridge; (**b**) deflection response of steel-cable-stayed bridge.

Figure 21 gives the flexural deflection responses of the bridge girder under moving traffic at speeds of 60 km/m, 80 km/m and 100 km/h. The horizontal axis represents the location of the vehicle, and the vertical axis represents the girder deflection. For both a CFRP cable-stayed bridge and a steel cable-stayed bridge, the deflection responses at mid-span are dominated by static deflection and a long-period vibration response, and the short-period vibration response is insignificant. The influence of traffic speeds was found to be very limited. Figure 21 shows that the flexural deflection of CFRP cable-stayed bridge is greater than that of the steel-cable-stayed bridge. This is because the CFRP cable-stayed bridge has relatively less structural rigidity, and the deflection response is mainly caused by static deformation.

**Figure 22:**
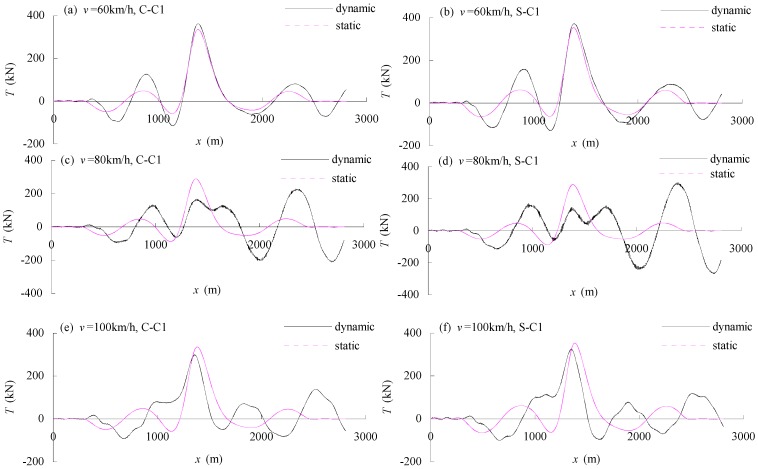
Static and dynamic tension responses of cables: (**a**) CFRP cable under vehicles moving at 60 km/h; (**b**) steel cable under vehicles moving at 60 km/h; (**c**) CFRP cable under vehicles moving at 80 km/h; (**d**) steel cable under vehicles moving at 80 km/h; (**e**) CFRP cable under vehicles moving at 100 km/h; (**f**) steel cable under vehicles moving at 100 km/h.

Figure 22 shows the static and dynamic tension responses of cable C1 of a cable-stayed bridge with moving traffic. The tension response of the CFRP cable was generally similar to that of the steel cable, even though the natural frequencies of CFRP cable and steel cable are different. This indicates that the longitudinal vibration of the cable is mainly caused by the vibrations of the support structure, *i.e.*, the anchorage points at the girder and the pylon. The local vibration of the cable does not influence this response.

## 6. Conclusions

To explore the feasibility of using CFRP cables in long-span cable-stayed bridges, this paper establishes a finite element model of a cable-stayed bridge with an ultimate span of 1400 m. The static and dynamic characteristics of cable-stayed bridges with CFRP cables are studied here. By comparing CFRP cables with steel cables in terms of mechanical behavior, some conclusions are drawn:

(1) For the same span cables in a 1,400 m span cable-stayed bridge, the self-weight of CFRP cable was only about 1/7 that of steel cable. Additionally, the Irvine parameters of CFRP cables are only about 1/6 that of steel cables. Therefore, the CFRP cable performance in tension is better than that of steel cables.
(2) For the same cable span, the natural frequencies of CFRP cables are about two times those of steel cables. Additionally, the optimal viscous damping coefficients of CFRP cables are about half those of steel cables. Therefore, the CFRP cables can help prevent the cable-bridge coupling vibration caused in lower modes of the cables. The attenuation of the parametric and wind fluctuating vibrations is better for CFRP as compared to the steel cables as suggested by the response amplitudes of the cables. The vibration amplitudes of CFRP cables are less than half those of steel cables.
(3) CFRP cable-stayed bridge has relatively less structural rigidity, so the maximum girder deflection of CFRP cable-stayed bridge is about 1.22-times the steel-cable-stayed bridge. However, the maximum compressive stress in the girders of CFRP-cable-stayed bridges is 9% lower than that of steel cable-stayed bridges. Additionally, the minimum stability factor of CFRP cable-stayed bridges is 5% larger than that of steel cable-stayed bridges.
(4) The CFRP cables have a lesser windward area than steel cables. Therefore, the transverse bending moment and out-of-plane deformation of mid-span girders is slightly larger for steel cable-stayed bridges than that for CFRP cable-stayed bridges.
(5) An analysis of vehicle-bridge coupling vibrations showed that replacing steel cables with CFRP cables has little impact on the vibration response of a cable-stayed bridge.

Using CFRP cables on long-span cable-stayed bridges is facilitatory in terms of mechanical properties. As the manufacturing process matures and the cost decreases, CFRP cables will see more use in long-span cable-stayed bridges. 
